# Diet and exercise advice and referrals for cancer survivors: an integrative review of medical and nursing perspectives

**DOI:** 10.1007/s00520-022-07152-w

**Published:** 2022-05-26

**Authors:** Ria Joseph, Nicolas H. Hart, Natalie Bradford, Oluwaseyifunmi Andi Agbejule, Bogda Koczwara, Alexandre Chan, Matthew P. Wallen, Raymond J. Chan

**Affiliations:** 1 Caring Futures Institute, College and Nursing and Health Sciences, Flinders University, Adelaide, SA Australia; 2 Cancer and Palliative Care Outcomes Centre, School of Nursing, Queensland University of Technology, Brisbane, QLD Australia; 3 Exercise Medicine Research Institute, School of Medical and Health Science, Edith Cowan University, Perth, WA Australia; 4 Institute for Health Research, The University of Notre Dame Australia, Perth, WA Australia; 5 Flinders Health and Medical Research Institute, College of Medicine and Public Health, Flinders University, Adelaide, SA Australia; 6 Department of Clinical Pharmacy Practice, School of Pharmacy and Pharmaceutical Sciences, University of California, Irvine, USA; 7 School of Science, Psychology and Sport, Federation University Australia, Ballarat, VIC Australia

**Keywords:** Health personnel, Cancer survivor, Attitude, Role, Diet, Exercise, Physical activity

## Abstract

**Purpose:**

To examine the perspectives of medical and nursing health professionals concerning their roles and responsibilities in providing dietary and exercise advice to cancer survivors, and referrals to allied health professionals.

**Methods:**

An integrative review. PubMed, CINAHL, PsycINFO, Embase, Web of Science databases, and bibliographies of relevant studies were searched from December 2011 to June 2021. All studies were eligible for inclusion. The Mixed-Methods Appraisal Tool (MMAT) was used to critically appraise included studies. Data were extracted and synthesised regarding the perspectives of medical and nursing health professionals on their roles, responsibilities, barriers, and facilitators.

**Results:**

Twenty-one studies involving 3401 medical and nursing health professionals and 264 cancer survivors of diverse cancer types were included. Ten quantitative, nine qualitative, and two mixed-methods studies were eligible. All included studies met at least 80% of the quality criteria in the MMAT. Major findings include the following: (1) medical and nursing health professionals were unclear on their roles in providing dietary and exercise advice to cancer survivors but agreed they play a key role in referrals to dietitians and exercise professionals; (2) most cancer survivors valued the involvement of their general practitioner when receiving dietary and exercise advice.

**Conclusion:**

Although medical and nursing health professionals understand that referrals to allied health professionals form part of their role, there is a lack of clarity regarding their roles to provide dietary and exercise advice to cancer survivors. Future studies should address barriers and facilitators of dietary and exercise advice and referral by medical and nursing health professionals.

**Supplementary Information:**

The online version contains supplementary material available at 10.1007/s00520-022-07152-w.

## Background

As the number of cancer survivors increases globally, there is growing recognition of the need to prevent and manage the numerous short and long-term challenges confronting them [1]. Cancer survivors often experience symptoms that have negative impacts on food intake, physical function, and body composition, during and after cancer treatment that often persists in the post-treatment phase [2–4]. Diet and exercise are well-established clinical interventions that can prevent, reduce, or reverse the physical and psychosocial effects of cancer and its treatment [5, 6].

Multidisciplinary teams are considered the “gold standard” of cancer care delivery worldwide, positively contributing to the improvement of cancer care and outcomes [7, 8]. Multiple studies have shown that a multidisciplinary approach incorporating medical, nursing, and allied health professionals supports cancer survivors during and after treatment to develop healthy lifestyle habits that improve clinical outcomes [9–11]. Allied health professionals, including dietitians and exercise professionals (e.g. clinical exercise physiologists and physiotherapists), work closely with medical and nursing professionals to provide expertise to prevent, diagnose, and treat various conditions and illnesses, inclusive of cancer [12]. In the survivorship context, dietitians provide individually tailored nutritional plans to optimise dietary intake and decrease nutrition-related side effects of cancer and its treatment [13]. Clinical exercise physiologists and physiotherapists are the most appropriate health professionals to promote physical activity and are tertiary-trained to design, prescribe, and deliver safe and effective exercise interventions to cancer survivors that optimise cardiorespiratory and neuromuscular fitness, body composition, and known cardiometabolic and musculoskeletal risk factors, thus improving cancer care and recovery [14, 15]. Accordingly, health professionals with a diversity of clinical specialties can play important synergistic roles in providing optimal dietary and exercise care to cancer survivors.

Medical and nursing health professionals are the most constant cancer care providers throughout a patient’s cancer journey, thereby playing an essential role in communicating evidence-based advice to cancer survivors, including for diet and exercise, as trusted agents for credible health information in cancer care [16, 17]. In this review, dietary and exercise advice includes (1) encouraging cancer survivors to adopt a healthy balanced diet; (2) increasing physical activity levels; (3) discussing the role of diet, physical activity, and structured exercise programs in cancer care and recovery; and (4) recommending adherence to diet and exercise guidelines [14, 18, 19]. This should be facilitated by providing cancer survivors with referrals to dietitians and exercise professionals with experience in cancer care for formal dietary and exercise assessments and interventions [20]. However, it is important to determine whether medical and nursing health professionals perceive this to be within their professional roles and responsibilities when caring for cancer survivors. Accordingly, the primary aim of this integrative review is to systematically harmonise existing perspectives of medical and nursing health professionals about their roles and responsibilities in providing dietary and exercise advice to cancer survivors, with referrals to allied health professionals; secondary aims of this integrative review were to identify barriers and facilitators to providing dietary and exercise advice and referrals by medical and nursing health professionals to cancer survivors.

## Methods

**Fig. 1:**
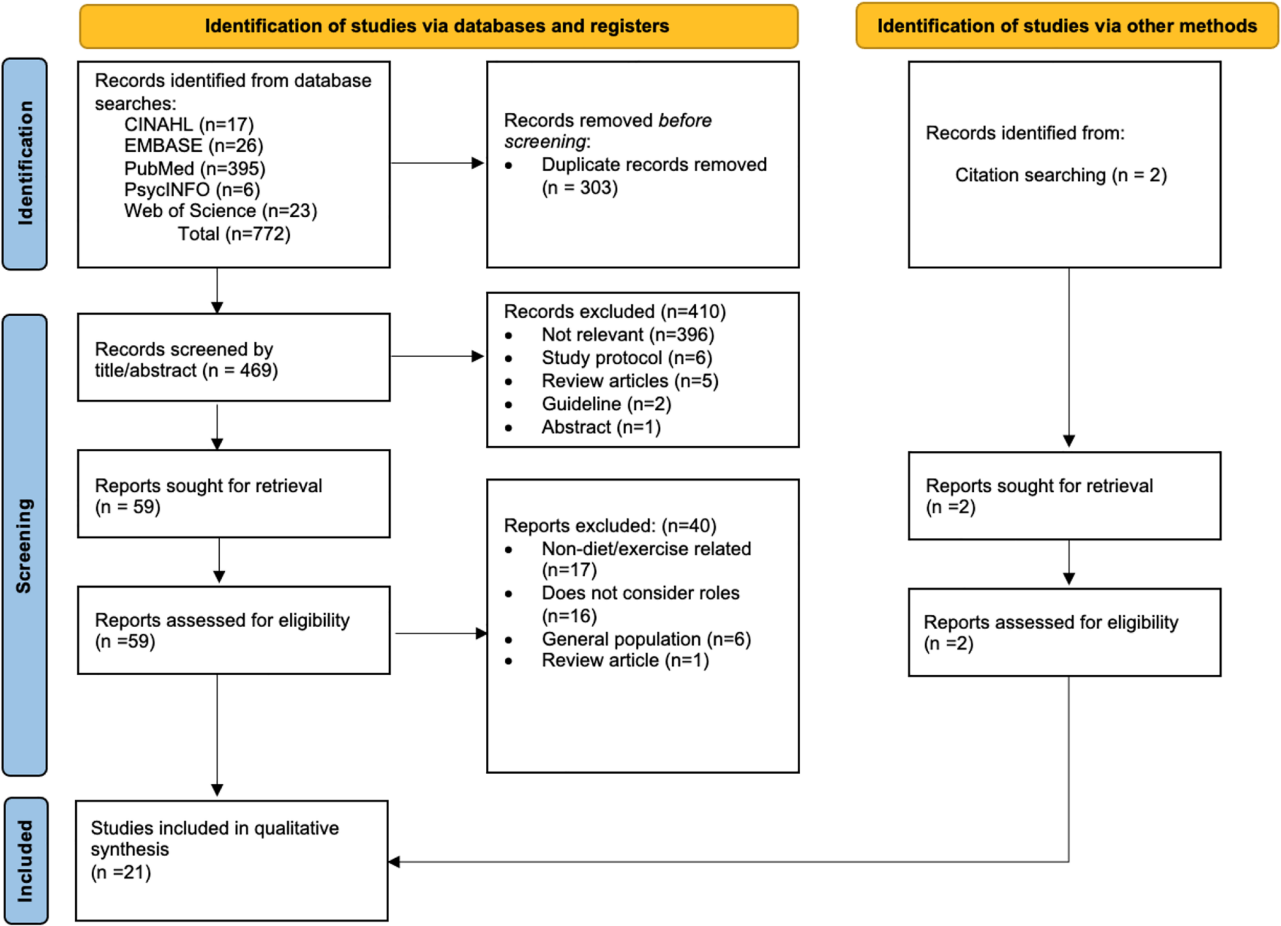
Flow diagram of literature search and selection process (PRISMA)

This is an integrative review involving studies of varied methodologies, including quantitative, qualitative, and mixed-methods research, unlike a systematic review that often focuses on a single research method (e.g. randomised controlled trials) or a narrow inquiry [21]. For this review, medical and nursing health professionals constitute cancer specialists, cancer nurses, and other generalists (GPs and practice nurses) who provide healthcare to cancer survivors. This integrative review adheres to the Preferred Reporting Items for Systematic Reviews and Meta-Analyses (PRISMA) checklist [22], with literature search and study selection processes illustrated (Fig. 1). The protocol of this review was registered at the International Prospective Register of Systematic Reviews (PROSPERO), registration number CRD42021225213.

### Selection criteria

Studies had to meet the following inclusion criteria: (1) published in peer-reviewed journals in English; (2) qualitative, quantitative, or mixed-methods study designs; (3) reporting perspectives of medical and nursing health professionals and consumers (i.e. cancer survivors and their families or informal caregivers) on the roles and responsibilities of medical and nursing health professionals; and (4) reporting the provision of dietary and exercise advice to cancer survivors. Studies reporting broader lifestyle advice that included diet, exercise, or both were also eligible.

### Search strategy

Articles were identified from December 2011 to June 2021 using a defined search strategy (Online Resource) across the following electronic databases: PubMed, CINAHL, PsycINFO, Embase, Web of Science; and hand searching of bibliographies of relevant studies. The search was limited to articles from 2011 to 2021 to ensure that our findings were most reflective of perspectives on medical and nursing health professionals’ roles and responsibilities in current healthcare settings due to the rising number of cancer survivors and evolving evidence in diet and exercise for cancer survivors. After obtaining all references, duplicates were excluded by using EndNote (Thomson Reuters, USA)[23].

### Study selection

Three authors (NB, OA, RJ) independently screened all articles identified in the search (titles and abstracts) for relevance using a web-based application: Rayyan [24], and those selected were subject to full-text assessment. Any discrepancies were resolved by consensus. One author (RJ) assessed the selected full-text articles against the eligibility criteria. All eligible articles were tabulated and included in the review.

### Data extraction

Two researchers (OA, RJ) independently extracted the data using a predefined data extraction form (Online Resource 2). A data extraction form was developed according to Joanna Briggs Institute (JBI) [25] and literature associated with cancer survivorship concepts or methods. For each study, the following data were extracted: study citation, study methods (e.g. semi-structured interviews, cross-sectional surveys), population characteristics (e.g. type of health professional), outcome measures (e.g. perspectives of medical and nursing health professionals), recommendation type (e.g. dietary and exercise advice), perceived role and responsibility of medical and/or nursing health professional, barriers and facilitators, study findings (including summarised themes, subthemes in qualitative studies, and conclusions), and the quality of evidence.

### Quality appraisal

The Mixed-Methods Appraisal Tool (MMAT, Version 2018) was used to critically appraise included studies. Critical appraisals were conducted by one researcher (RJ). For qualitative studies, five questions appraised the methodological quality of the articles in terms of the following aspects: data sources, collection and analysis, context, and researchers’ influence on the data. Quantitative descriptive studies were appraised by five questions regarding the sampling strategy, representativeness, appropriateness of measurements, and response rate. For mixed-methods studies, five questions appraised the rationale for using a mixed-methods design, integration of qualitative and quantitative data, and discrepancies between qualitative and quantitative results (Table 1).

**Table 1:** Critical appraisals using the Mixed-Methods Appraisal Tool (MMAT**)**

Studies	Methodological quality criteria					Overall responses from the Mixed-Methods Appraisal Tool	Quality
Qualitative	1	2	3	4	5		
Baker, 2015	✓	✓	✓	✓	✓	QUAL = 5	100%
Balneaves, 2020	✓	✓	✓	✓	✓	QUAL = 5	100%
Brandenbarg, 2017	✓	✓	✓	✓	✓	QUAL = 5	100%
Cheville, 2012	✓	✓	✓	✓	✓	QUAL = 5	100%
Coa, 2014	✓	✓	✓	✓	✓	QUAL = 5	100%
Haussman, 2018a	✓	✓	✓	✓	✓	QUAL = 5	100%
Koutoukidis, 2018	✓	✓	✓	✓	✓	QUAL = 5	100%
Roberts, 2019	✓	✓	✓	✓	✓	QUAL = 5	100%
Waterland, 2020	✓	✓	✓	✓	✓	QUAL = 5	100%
Quantitative descriptive	1	2	3	4	5		
Alderman, 2020	✓	✓	✓	x	✓	QUAN = 4	80%
Chan, 2018	✓	✓	✓	x	✓	QUAN = 4	80%
Haussman, 2018b	✓	✓	✓	x	✓	QUAN = 4	80%
Keogh, 2017	✓	✓	✓	x	✓	QUAN = 4	80%
Kiss, 2020	✓	✓	✓	x	✓	QUAN = 4	80%
Ligibel, 2019	✓	✓	✓	x	✓	QUAN = 4	80%
Puhringer, 2015	✓	✓	✓	x	✓	QUAN = 4	80%
Spellmann, 2014	✓	✓	✓	x	✓	QUAN = 4	80%
Wallace, 2015	✓	✓	✓	x	✓	QUAN = 4	80%
Williams, 2013	✓	✓	✓	x	✓	QUAN = 4	80%
Mixed-methods							
Anderson, 2013	✓	✓	✓	x	✓	MM = 4	80%
Kassianos, 2017	✓	✓	✓	x	✓	MM = 4	80%

### Data synthesis and analysis

Extracted data were analysed by one author (RJ) according to the stages described by Whittemore and Knafl [21], using a mixed-methods synthesis [26]. This process was checked by co-supervisors (RJC, NHH, and NB). Firstly, a segregated synthesis analysis was performed on each study design. Data reduction was performed on categories that were relevant to the review questions. The next step was data display, converting data from individual sources into a matrix display to assemble data across all study designs. Data comparisons between the matrix displays resulted in subthemes and main emerging themes. The emerging themes were categorised into a table format. Quantitative data were then translated to qualitative data using a convergent integrated approach, which involves data transformation for a mixed-methods synthesis [27]. This involved conversion into narrative interpretations of quantitative results from all studies (including quantitative portions of mixed-methods studies). Due to the heterogeneity of included studies, extracted data from quantitative and qualitative studies were combined according to JBI methodology for mixed-methods systematic review [26]. This involved assembling the converted quantitative data with the qualitative data. Assembled data were categorised and synthesised based on similarity in meaning to produce a set of integrated findings.

## Results

The search strategy yielded 772 articles in which 303 duplicates were identified and removed. After screening titles and abstracts, 61 articles were selected for full-text review. Forty articles were excluded following full-text review as they did not meet the inclusion criteria. Ultimately, 21 articles (10 quantitative, 9 qualitative, and 2 mixed-methods) met the inclusion criteria and were subjected to quality appraisal (Fig. 1).

### Study characteristics

**Fig. 2:**
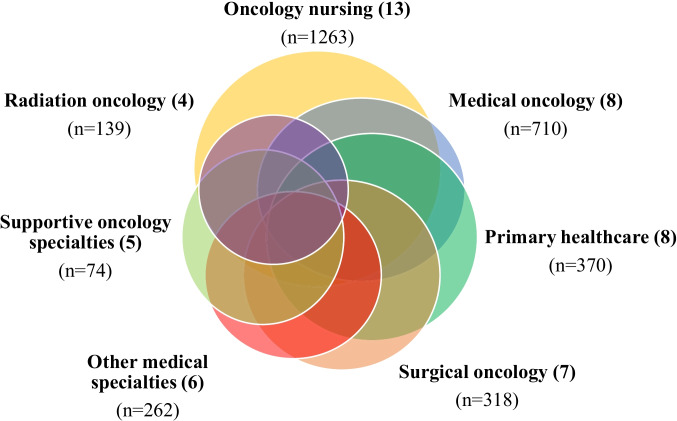
Distinct specialities across included studies (*n* = number of participants)

Of the 21 articles, 16 included medical and nursing health professionals, three included cancer survivors, and two included both, totalling 3401 medical and nursing health professionals and 264 consumers with diverse cancer types (Fig. 2). The nine qualitative studies explored the perspectives of 167 medical and nursing health professionals and 54 cancer survivors. These were conducted in the USA (*n* = 3) [28–30], UK (*n* = 2) [1, 31], Australia (*n* = 1) [32], Canada (*n* = 1) [9], Germany (*n* = 1) [33], and the Netherlands (*n* = 1) [34]. Of the 10 quantitative studies, 2812 health professionals and 222 cancer survivors were surveyed across Australia (*n* = 5) [35–39], Australia and New Zealand (*n* = 2) [40, 41], Germany (*n* = 1) [42], UK (*n* = 1) [17], and USA (*n* = 1)[43] about their perspectives. Two mixed-methods studies were conducted in the UK (*n* = 2)[44, 45] and explored the perspectives of 418 medical and nursing health professionals. Overall, the total sample included a mixture of professions, including cancer nurses, general practitioners (GPs), and cancer specialists (i.e. oncologists, medical oncologists, radiation oncologists, surgeons, and urologists), as well as consumers (i.e. cancer survivors, families, and caregivers). The following interventions were reported: general dietary and exercise advice, promotion of changes to diet and physical activity, and the provision of weight management advice.

### Methodological quality of the studies

All nine qualitative studies met 100% of the quality criteria, with no obvious methodological limitations as appraised using the MMAT. In addition, amongst the 10 quantitative and 2 mixed-methods studies, all met 80% of the quality criteria. Methodological limitations such as unclear response rates, in particular nonresponse bias, was identified in 12 studies.

### Main themes

**Fig. 3:**
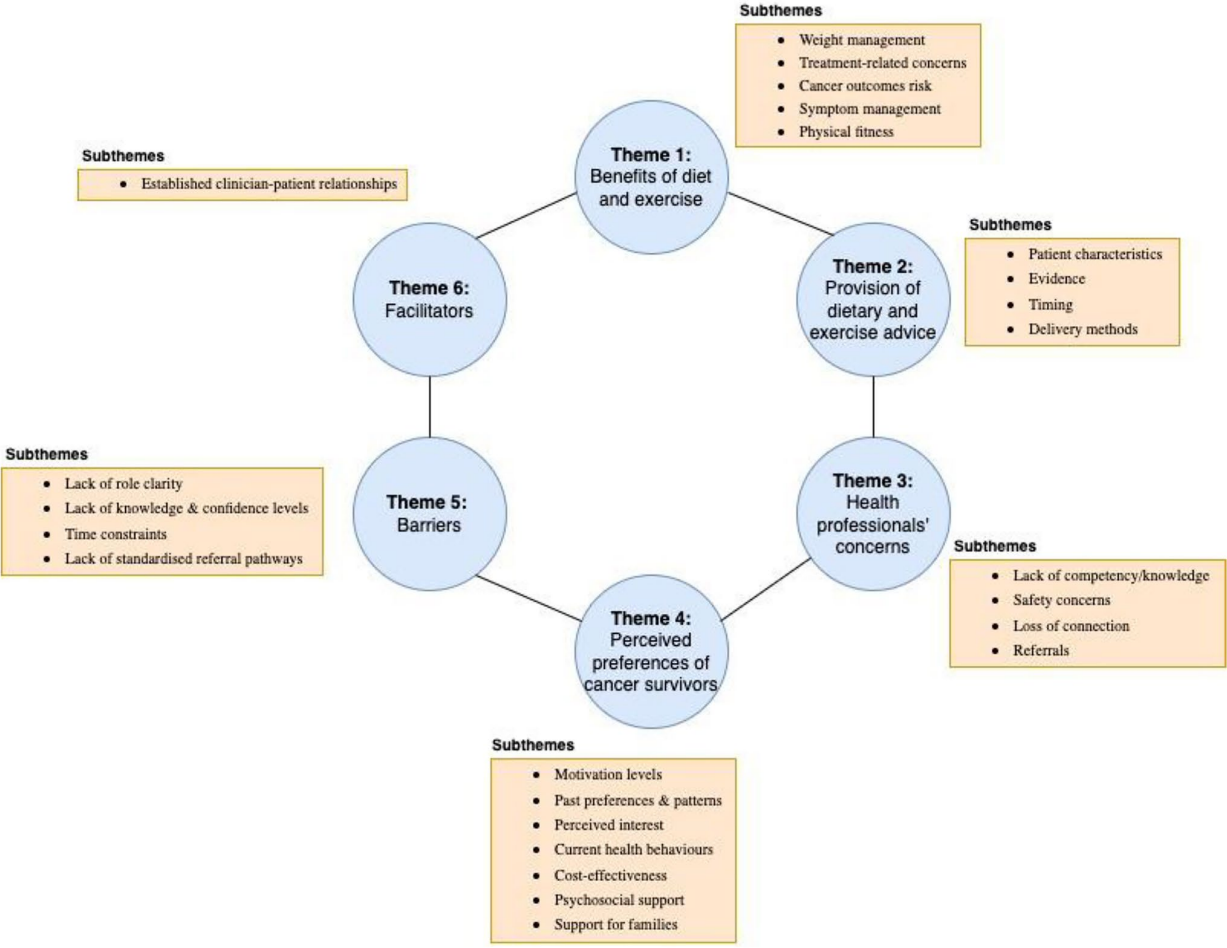
Themes and subthemes of included studies

Six themes were identified in the analysis and subsequently synthesised: (1) benefits of diet and exercise; (2) provision of dietary or exercise advice; (3) health professionals’ concerns for providing dietary or exercise advice; (4) perceived preferences of cancer survivors; (5) barriers; and (6) facilitators to providing dietary or exercise advice and referrals by medical and nursing health professionals (Fig. 3).

### *Roles and responsibilitiesof medical and nursing health professionals*

#### Benefits of diet and exercise

Medical and nursing health professionals acknowledge that diet and exercise play an important role in maintaining health while improving clinical outcomes for cancer survivors, and that dietary and exercise support form part of a multidisciplinary approach to cancer care [5, 6, 45]. It is of critical importance that medical and nursing health professionals convey and promote this message to cancer survivors [45, 46].

#### Provision of dietary and exercise advice

Most medical and nursing health professionals perceived their role to include the provision of regular advice to cancer survivors to achieve or maintain a healthy weight, increase physical activity, and eat a healthy and balanced diet across multiple treatment stages [28, 43]. Some medical and nursing health professionals perceived their role to include empowering cancer survivors to adopt a healthier lifestyle by recommending small, achievable changes to their diet or physical activity behaviour [28, 31]. The provision of dietary and exercise advice was dependent on multiple factors, including patient preferences, strength of the evidence, optimal timing, and delivery methods. Medical and nursing health professionals highlighted that tailoring advice on diet, physical activity, or exercise should consider the cancer survivor’s cognitive ability, relationship with their health professionals, physical condition, and overall well-being [31, 32, 45]. Cancer survivors’ current health behaviours; perceived interest in diet, physical activity, or exercise; motivation levels; and past preferences and patterns were also considered [1]. However, medical and nursing health professionals had varying perspectives on when cancer survivors were most motivated to make changes. Several suggested the optimal timing to provide dietary and exercise advice should be provided at diagnosis or during treatment and re-enforced at each follow-up appointment to enact meaningful health behaviour changes [30]. As a result, cancer survivors could establish, maintain, or progress habitual dietary, physical activity, and exercise behaviours that would help prevent, reduce, or reverse side effects relating to treatment, and prevent potential weight gain [9, 41]. Other medical and nursing health professionals thought dietary and exercise advice should wait until after treatment because the treatment itself is a big adjustment for cancer survivors (e.g. physical, and psychosocial effects of treatment) [30]. Overall, medical and nursing health professionals believed they should provide dietary or exercise advice to cancer survivors at key time points when they are motivated and likely to make changes.

#### Health professionals’ concerns

Some medical and nursing health professionals did not consider themselves as competent, responsible, or appropriate to provide dietary and exercise advice to cancer survivors, given their limited knowledge of the topic, their clinical role, training, or other higher priorities during short consultations [28, 31, 33, 38]. Medical health professionals have reported uncertainty with what to recommend, and fears of potential safety risks to cancer survivors, such as physical overexertion and psychological stress during physical activity or exercise [1, 42]. Other concerns reported included potential loss of connection with the cancer survivor, and fear of potentially setting expectations that may not be achievable as cancer survivors’ goals can change during cancer treatment [1]. In contrast to medical health professionals, cancer nurses had fewer concerns and were more likely to discuss diet, physical activity, and exercise with cancer survivors [1, 36, 39, 40]. Overall, these studies highlighted that there is variability in medical and nursing health professionals’ views on their ability to provide dietary and exercise advice.

#### Perceived preferences of cancer survivors

Despite growing evidence that dietary and exercise health behaviours are linked to improved outcomes in cancer survivors [47], few cancer survivors report receiving diet and exercise advice from their health professionals [46], whereby some cancer survivors reported this lack of encouragement as a major reason for not committing to engaging in a healthy diet or more physical activity [29]. Cancer survivors emphasised the valuable role of GPs in their follow-up care, including the provision of significant support for managing the physical and psychological effects of cancer and its treatment and health promotion [17, 34, 45]. In addition, cancer survivors welcomed guidance on diet and exercise, as well as referral to dietitians and exercise professionals, particularly in the immediate post-treatment phase, because of the uncertainties of cancer and treatment-related side effects [34]. GP care was not limited to the cancer survivors alone; relatives also consulted the GP for issues about coping with cancer in the family. This was highly valued and provided security for cancer survivors knowing family members were also cared for [34]. Several cancer survivors highlighted a preference to receive exercise guidance from their cancer specialist who provided general encouragement for cancer survivors to “stay active”, while others indicated they were unsure whether to seek advice from medical or nursing health professionals on this topic [29]. In contrast, some cancer survivors had no preferences to which specific health professional provided dietary or exercise advice, so long as it is received from a professional with whom they have a trusting relationship [34]. No studies in this review included nursing health professionals, thus the perceived preferences of cancer survivors for nurses as valuable agents of diet and exercise advice, and referrals to allied health need to be explored in future research.

### Barriers and facilitators

#### Barriers to providing dietary and exercise advice, and referrals to allied health

##### ***Lack of role clarity***

Medical and nursing health professionals had different views regarding their perceived roles, with a lack of clarity observed. Even though most cancer nurses and GPs perceived their roles to involve the discussion of diet, physical activity, and exercise [1, 32, 35, 36, 39–41, 45], some did not perceive it to be part of their role given their limited knowledge on the topic and other priorities during patient interactions [31, 48, 49]. Cancer specialists felt they were less likely to provide dietary and exercise advice than primary care providers, as they believed it was not their role and that cancer survivors engaged with primary care for surveillance [30]. However, cancer specialists did perceive their role to include providing referrals to other health professionals or community programs [9]. There was a preference amongst medical and nursing health professionals to refer cancer survivors to allied health professionals [33], with GPs preferring a dietitian or exercise professional to be the primary care provider responsible for providing dietary and exercise advice [35]. Therefore, cancer nurses and GPs may perceive their roles to be different from cancer specialists with regard to providing dietary and exercise advice, but they all perceive referral to be part of their role.

##### Lack of knowledge and confidence

Medical and nursing health professionals recognise that positive changes in diet and exercise in cancer survivors are likely beneficial; however, few receive training within their curriculum on the importance of diet, physical activity, or exercise [28, 35, 38, 43, 44]. As a result, medical and nursing health professionals report a lack of confidence in providing appropriate advice and are less likely to provide information on a healthy diet and physical activity to cancer survivors [33, 45].

##### ***Time constraints***

Medical and nursing health professionals all reported that a common and considerable barrier to promoting healthy eating, physical activity, and exercise for cancer survivors is a lack of time during consultations to discuss dietary and exercise advice [1, 9, 32].

##### Lack of standardised referral pathways

Medical and nursing health professionals report assessing dietary patterns and physical activity levels during and after cancer treatment, and providing general dietary, physical activity, and weight management advice. However, this often did not result in referrals to allied health professionals or diet and exercise specialists to enact and support lifestyle change [43]. This could be attributed to limited guidance and little information available to medical and nursing health professionals, as there is currently no standardised referral framework and no structured referral process for them to adopt. Therefore, there is a need to provide information on how, and when, to refer cancer survivors, inclusive of the necessary clinical information required to standardise and streamline the process, and connectivity to these services.

#### Facilitators to providing dietary and exercise advice and relevant referrals

##### Established clinician-patient relationships

Cancer survivors are under the routine care of cancer specialists, and cancer nurses throughout the cancer continuum, which enables an established and trusted relationship [1]. Cancer nurses have the most frequent time and opportunity to interact with cancer survivors providing greater opportunities to intervene, educate, and refer to supportive care services [40]. GPs can also contribute to increasing the referral patterns to specialists due to their positive attitudes toward, and knowledge of the benefits of diet and exercise for cancer survivors [32, 35]. Therefore, medical and nursing health professionals are well-positioned to inform cancer survivors about diet and exercise as a trusted source.

## Discussion

Most medical and nursing health professionals consider diet and exercise interventions beneficial to cancer survivors according to the results of our integrative review, consistent with earlier evidence [50]. Even though there is variability of views on their roles and responsibilities in providing advice on diet, physical activity, or exercise to influence behaviour change in cancer survivors [30], there was overall confidence that their role should be to provide referrals to dietitians and exercise professionals [33, 35, 40]. This suggests that there is a lack of clarity between medical and nursing health professionals regarding their roles and responsibilities in providing dietary and exercise advice. This could be attributed to several factors, including poorly defined roles. Clarifying professional roles can ensure the appropriate implementation of medical and nursing health professionals’ role regarding dietary and exercise advice to cancer survivors and therefore ensure better care of the cancer survivor [51, 52]. This could be achieved by using a consensus process to facilitate role clarity and establish consensus between professional groups [53]. Better guidance is required for medical and nursing health professionals, in terms of what advice they should provide and when to provide the advice, as well as how and when to refer cancer survivors to dietitians and exercise professionals.

Medical and nursing health professionals identified that providing dietary and exercise advice to cancer survivors remains an ongoing issue due to various health professional and patient-centred barriers. As a result, additional training is required to assist medical and nursing health professionals to provide physical activity, adequate weight management and dietary and exercise advice as part of routine practice, and resources [35, 44]. This may include continuing professional development (CPD) for medical and nursing health professionals where this type of training could be offered [54]. It is important that these programs and opportunities are evaluated, with outcomes addressing the reaction, learning, behaviour, and results levels [55]. Embedding such additional training within university programs has the potential to improve their dietary and exercise management skills and confidence levels that can positively impact patient health outcomes [56–58]. Ideally, resources should include clear information on specific dietitians and exercise professionals who can provide proper assessment, programming, and support; and information on referral pathways to reach these specialists or community services [42]. However, significant barriers to undertaking CPD and training at universities exist, including a lack of time, understaffing, competition for time in the existing curriculum, and perceived importance at the faculty level in universities [58, 59]

Many cancer survivors have reported high levels of trust in their usual GP, and hence value the involvement of their GPs for their dietary and exercise advice and referrals [34]. Therefore, cancer survivors may be more likely to follow their GP’s advice, resulting in more beneficial health behaviours. Not only are GPs integral in cancer detection as the first point of contact for cancer survivors, but they are also able to provide care and key referrals in the post-treatment follow-up phase. This can include ongoing support and follow-up to assist the maintenance of lifestyle changes [15, 59]. Furthermore, many GPs have established relationships with cancer survivors, so they are well placed to support them (i.e. providing general lifestyle-related advice) [32]. Since GPs can play a key role in counselling cancer survivors about diet and physical activity or exercise, it is vital that they are provided with proper resources, training, and support throughout the cancer care continuum.

### Limitations and strengths

This review has several limitations. While a strength of this review pertains to the global nature of studies included, these were all from Western countries that were high-income; thus, the findings of this review may not reflect those of other cultures, or those that are low-to-middle income countries with different health system priorities. Cancer survivors in various settings have been included in this review; however, these studies did not define the diagnoses and care context. Accordingly, not all findings are generalisable to all settings and health systems. Nevertheless, the major strength of this review is that it addresses the perspectives of medical health professionals, nursing health professionals, and cancer consumers (cancer survivors, families, and caregivers). Another strength is the integrated review method, which facilitates the inclusion and integration of different sources and types of information in a single review.

### Implications for research and practice

Given the lack of representation from low-to-middle income countries in this review, future research is required to reflect the perspectives of the larger group more accurately. Additionally, emphasis should be placed on providing better guidance and structure to medical and nursing health professionals by clearly defining the roles they play in providing dietary and exercise advice, and by clearly defining the process of how and when to refer cancer survivors to diet and exercise professionals. Therefore, future research should focus on the development of standards of care for optimal referral practices to dietary and exercise services to facilitate role clarity and promote consistent cancer care provision between medical and nursing health professionals. The review highlights some clinical implications that could benefit from further research, including addressing the specific barriers to the provision of dietary and exercise advice to cancer survivors and further identifying barriers and facilitators to referral to relevant allied health professionals.

## Conclusion

Some, but not all, medical and nursing health professionals provide generic dietary and exercise advice to cancer survivors based on their individual needs, preferences, or strength of available evidence. Although medical and nursing health professionals understand, and agree, that referring cancer survivors to allied health professionals form part of their role, there is a lack of clarity between medical and nursing health professionals regarding roles and responsibilities in providing dietary and exercise advice to cancer survivors. Furthermore, even though barriers and facilitators to providing dietary and exercise advice have been identified, there is limited evidence on barriers and facilitators specific to referral practices, which require further research.

## Supplementary Information

Below is the link to the electronic supplementary material.

Supplementary file1 (PDF 73 KB)

Supplementary file2 (PDF 182 KB)

## Data Availability

The authors confirm that the data supporting the findings of this study are available within the article and its supplementary materials.
