# Supplementary material for: Diet and exercise advice and referrals for cancer survivors: an integrative review of medical and nursing perspectives
Source: Support Care Cancer. 2022 May 26;30(10):8429–39. doi: 10.1007/s00520-022-07152-w (PMC9512858; doi:10.1007/s00520-022-07152-w)
Supplement: Supplementary file 1 — Supplementary file1 (PDF 73 KB) [file 520_2022_7152_MOESM1_ESM.pdf]

Journal: Supportive Care in Cancer

Article type: Review Article

Title: Diet and exercise advice and referrals for cancer survivors: an integrative review of medical and nursing perspectives.

Ria Joseph[1*], Nicolas H. Hart[1,2,3,4], Natalie Bradford[2,], Oluwaseyifunmi Andi Agbejule[1], Bogda Koczwara[5], Alexandre Chan[6], Matthew P. Wallen[1,7], Raymond J. Chan[1,2]

1. Caring Futures Institute, College and Nursing and Health Sciences, Flinders University, Adelaide, SA, Australia.
2. Cancer and Palliative Care Outcomes Centre, School of Nursing, Queensland University of Technology, Brisbane, QLD, Australia.
3. Exercise Medicine Research Institute, School of Medical and Health Science, Edith Cowan University, Perth, WA, Australia
4. Institute for Health Research, The University of Notre Dame Australia, Perth, WA, Australia
5. Flinders Health and Medical Research Institute, College of Medicine and Public Health, Flinders University, Adelaide, SA, Australia.
6. Department of Clinical Pharmacy Practice, School of Pharmacy and Pharmaceutical Sciences, University of California, Irvine USA
7. School of Science, Psychology and Sport, Federation University Australia, Victoria, Australia

[*] **Corresponding author:**

Ms Ria Joseph, PhD (cand.), APD.

Caring Futures Institute, College of Nursing and Health Sciences, Flinders University

Email: ria.joseph@flinders.edu.au; Telephone: +61 406 437 648.

Online Resource

Table 1 Search Strategy

Stages	Search terms and keywords (PubMed)
Stage 1 (Initial search)	(perspective* OR view* OR perception* OR attitude* OR belief*) AND ("general practitioner*" OR GP* OR nurse* OR oncologist* OR doctor* OR physician*) AND (role*) AND (diet OR nutrition OR "healthy eating" OR exercise OR "physical activity" OR activity OR training OR lifestyle OR "lifestyle advice" OR "health promotion") AND (consumer* OR patient* OR “cancer survivor*” OR cancer)
Stage 2	Hand searching of bibliographies of relevant studies
