# Supplementary material for: Diet and exercise advice and referrals for cancer survivors: an integrative review of medical and nursing perspectives
Source: Support Care Cancer. 2022 May 26;30(10):8429–39. doi: 10.1007/s00520-022-07152-w (PMC9512858; doi:10.1007/s00520-022-07152-w)
Supplement: Supplementary file 2 — Supplementary file2 (PDF 182 KB) [file 520_2022_7152_MOESM2_ESM.pdf]

Journal: Supportive Care in Cancer

Article type: Review Article

Title: Diet and exercise advice and referrals for cancer survivors: an integrative review of medical and nursing perspectives.

Ria Joseph[1*], Nicolas H. Hart[1,2,3,4], Natalie Bradford[2,], Oluwaseyifunmi A. Agbejule[1], Bogda Koczwara[5], Alexandre Chan[6], Matthew P. Wallen[1,7], Raymond J. Chan[1,2]

1. Caring Futures Institute, College and Nursing and Health Sciences, Flinders University, Adelaide, SA, Australia.
2. Cancer and Palliative Care Outcomes Centre, School of Nursing, Queensland University of Technology, Brisbane, QLD, Australia.
3. Exercise Medicine Research Institute, School of Medical and Health Science, Edith Cowan University, Perth, WA, Australia
4. Institute for Health Research, The University of Notre Dame Australia, Perth, WA, Australia
5. Flinders Health and Medical Research Institute, College of Medicine and Public Health, Flinders University, Adelaide, SA, Australia.
6. Department of Clinical Pharmacy Practice, School of Pharmacy and Pharmaceutical Sciences, University of California, Irvine USA
7. School of Science, Psychology and Sport, Federation University Australia, Victoria, Australia

[*] **Corresponding author:**

Ms Ria Joseph, PhD (cand.), APD.

Caring Futures Institute, College of Nursing and Health Sciences, Flinders University

Email: ria.joseph@flinders.edu.au; Telephone: +61 406 437 648.

Online Resource 2

Table 2 Data extraction table

Reference citation/ Country	Study Type/ Methods	Population	Outcome measures	Recommendation type	Sample size	Findings	Perceived Role of HP	Barriers/Facilitators	Summary of findings/ Conclusions	Themes	Quality
**Qualitative studies**											
Baker et al., 2015/ USA	Semi-structured interviews	Oncologists, surgeons, primary care providers, nurses, dietitians working with prostate, breast, or non-Hodgkin lymphoma cancer survivors.	Body size (BMI) and weight management.<br><br>Achieving a healthy weight.	Weight management	n=33	Most HPs focused on weight gain as opposed to weight loss.<br><br>Providers described lacking weight loss strategies to implement without referrals.<br><br>Some practitioners reported a hands-on approach in the office, using educational handouts, such as recipe books and portion guides, and graphs that help cancer survivors visualize information<br><br>Variation in providers' reported exercise recommendations, with some questioning the utility of exercise for weight loss,	Some providers reported reducing weight discussions as they do not feel it is appropriate for their clinical role or training.<br><br>However, PCPs reported frequent weight- related discussions, and cancer specialists also reported discussing weight management.	1.Perceived issues with training as established guidelines do not seem actionable to health professionals with limited experience in behavioural modification.<br><br>2.Lack of relevance to clinical roles.<br><br>3.Time constraints.	Health professionals can play an important role in providing weight management advice to cancer survivors. Given they are provided with sufficient resources and support.<br><br>Evidence-based clinical resources for weight management are required to provide appropriate advice to cancer survivors.	3 categories with 4 themes and 5 subthemes: benefits of diet/exercise, provision of dietary/physical activity advice, patient preferences, and barriers.<br><br>• Weight management<br>• Treatment-related concerns<br>• Cancer outcomes risk<br>• Timing<br>• Lack of relevance to clinical role	QUAL= 5<br>100%
Balneaves et al.,	Semi-structured interviews	Breast cancer survivors, and oncologists	Perceptions and experiences of providing	Aerobic and resistance training and	Breast cancer	Cancer survivors did not have	1.Time constraints	Cancer survivors require support during but also after completion of treatment to adopt healthy lifestyle		3 categories with 4 themes and 6 subthemes:	QUAL= 5<br>100%

2020/ Canada			lifestyle advice to cancer survivors (i.e., barriers and facilitators, their role)	nutrition counselling session.	survivors: n=12<br><br>Oncologists: n=8	preferences to which specific health professional provided lifestyle advice, except that they had a trusting relationship with them.<br><br>Oncologists and nurses can play a role in discussing the importance and safety of lifestyle interventions during breast cancer treatment. As well as encouraging healthy lifestyle behaviours and providing referrals to other health professionals or community programs.	2.No streamlined process to support health professionals in referring cancer survivors to lifestyle interventions.	habits. Oncologists play an important role in encouraging participation in lifestyle interventions during breast cancer treatment.<br><br>Maintenance programs that transition cancer survivors into community settings and provide on-going information and follow-up are also required.	benefits of diet/exercise, challenges, patient preferences, provision of dietary/physical activity advice.<br><br>• Motivation<br>• Symptom management<br>• Physical fitness<br>• Social support<br>• Trusting relationship<br>• Dietary/exercise advice	
Brandenbarg et al., 2017/ Netherlands	Semi-structured interviews	Colorectal cancer survivors	Role of GP during treatment.<br><br>Role of GP during follow-up	Dietary and exercise advice	n=22	GPs are perceived to play a role of providing guidance on diet and lifestyle in the immediate post-treatment period.<br><br>Cancer survivors reported that clarification offered by the	Participants often feel uncertain regarding their lifestyles as it may have not been discussed in secondary care.	Participants prefer to receive guidance on diet and lifestyle in the immediate post-treatment period.<br><br>Participants value the involvement of GPs in monitoring directly after surgery.	4 categories with 2 themes and 3 subthemes: provision of dietary/physical activity advice, patient preferences.<br><br>• Clarification<br>• Psychosocial support<br>• Support for families	QUAL= 5<br>100%

						GPs had a reassuring effect.				
Cheville et al., 2012/ USA	Semi-structured interviews	Cancer survivors with late-stage lung cancer.	Cancer survivors' views and preferences about exercise	Exercise guidance	n=20	Participants stated that they would be comfortable discussing exercise with their oncologist; however, few had done so.<br><br>Participants stated that receiving encouragement or an exercise prescription from their oncologist would give them the motivation to enhance their physical activity<br><br>Participants only received general encouragement to "stay active" from their oncologist.<br><br>Participants prefer exercise guidance from their oncologists as opposed to a physical therapist.<br><br>Opinions around exercise guidance from	1.Symptoms affecting their activity.<br><br>2.Past preferences and patterns<br><br>3.Lack of equipment<br><br>4.Differing views on being "exercise" or "non-exercise" people.<br><br>5.Lack of awareness on the importance of exercise.<br><br>6.Lack of encouragement from oncologists.	Exercise and physical activity can improve cancer-related symptoms (i.e., home programs that incorporate cancer survivors' daily activities).<br><br>Participants require encouragement and support from their oncologist for positive outcomes.	3 themes with 4 subthemes: provision of dietary/physical activity advice, patient preferences, and barriers.<br><br>• Motivation<br>• Symptoms<br>• Past preferences and patterns<br>• Lack of awareness	QUAL= 5 100%

						physical therapists varied with some participants believing that such assistance was for individuals with more functional disabilities and others viewing it as potentially beneficial.					
Coa et al., 2014/<br>USA	Interviews	Oncologists, surgeons, primary care providers, nurses, social workers, a registered dietitian, cancer survivors, navigator, and a survivorship coordinator who care for individuals diagnosed with breast cancer, prostate cancer, and non-Hodgkin's lymphoma.	Providers' roles and responsibilities in caring for long-term cancer survivors.<br><br>Perceived importance of behaviour changes and healthy diet for cancer survivors.<br><br>Barriers and facilitators.	Promote dietary changes	n=33	HPs generally agreed that making healthy dietary changes was good for the overall health of cancer survivors.<br><br>Variance among health professionals on the benefit of lifestyle changes - some believed the evidence to make lifestyle change recommendations was strong, others believed the evidence was not strong<br><br>Some HPs believed the evidence	Discussing healthy dietary behaviour changes with cancer survivors.	1. Strength of evidence was considered a priority to assess the importance of behaviour change among cancer survivors.<br><br>Health care providers were less likely to bring up diet if they perceived the evidence to be weak.	Health care providers can play an important role in promoting healthy diet among cancer survivors (i.e., incorporating behaviour changes into survivor care plans).	4 categories with 4 themes and 3 subthemes:<br>benefits of diet, perceived role of HPs, patient preferences, and barriers.<br><br>• Strength of evidence<br>• Timing<br>• Motivation	QUAL=<br>5<br>100%

						between diet and recurrence was inconclusive.<br><br>Specialist providers (e.g., oncologists, surgeons) expressed they were less likely to discuss health promotion than primary care providers, as they believed cancer survivors engaged with them for surveillance.					
Haussmann et al., 2018a/ Germany	Semi-structured interviews	GPs, specialised physicians, oncology nurses.	Knowledge<br><br>Perceived advantages and disadvantages of physical activity by cancer survivors.<br><br>Barriers and facilitators for promoting physical activity.	Physical activity promotion	n=30	Most health professionals did not consider their role to involve encouraging sedentary cancer survivors to make behavioural changes.<br><br>There was a preference to refer cancer survivors to physiotherapists, exercise counselling centres or recommend local exercise programs.	1.Health professionals valued a good relationship with their patient so preferred not to advise cancer survivors on how to spend their free time.<br><br>2.Time constraints so physical activity was not considered a priority (e.g., increased workloads).<br><br>3.Lack of knowledge in how to promote physical activity.<br><br>4.Lack of informative	A patient’s physical condition and assumed interest in physical activity is one of the main factors influencing if physical activity is addressed.<br><br>Patient characteristics and structural conditions for health professionals can influence physical activity promotion.<br>A broader perspective is needed to assess these factors.		3 themes with 5 subthemes:<br>HP’s concerns, patient preferences, and barriers.<br><br>• Physical overexertion<br>• Psychological stress<br>• Perceived interest in PA<br>• Current health behaviours<br>• HP’s workload	QUAL= 5<br>100%

							resources (e.g., brochures for both HCPs and cancer survivors)<br><br>5.Fear of physical overexertion and psychological stress for cancer survivors.				
Koutoukidis et al., 2018/ UK	Semi-structured interviews	Surgeons, physicians, nurses and allied health workers caring for those with breast, prostate, or colorectal cancer.	Survivorship-centred barriers to provision.<br><br>HP-centred barriers to provision.<br><br>Optimal delivery of lifestyle advice.	Dietary/exercise advice	n=21	Some health professionals perceived their role to include empowering cancer survivors to follow a healthier lifestyle.<br><br>Others did not perceive it to be part of their role given their limited knowledge on the topic and other priorities during their short consultations.	1.Limited knowledge on the topic and limited knowledge of healthy lifestyle guidelines.<br><br>2.Socioeconomic barriers and ability to practise health behaviours.<br><br>3.Fear for potential loss of connection with cancer survivors.<br><br>4.Time constraints (i.e., other priorities during short consultations).	Health professionals believed that dietary/exercise advice should be tailored to each individual and delivered throughout the cancer journey.<br><br>They also believed they should focus on small and achievable changes and be cost-effective.<br><br>When developing training programmes for health professionals, barriers need to be incorporated.<br><br>This will ensure successful behavioural changes and improve outcomes for cancer survivors.		4 themes with 6 subthemes: patient preferences, provision of dietary/physical activity advice, barriers and facilitators.<br>• Current health behaviours<br>• Physical inability<br>• Loss of connection with patient<br>• Socioeconomic barriers<br>• Knowledge & attitudes towards evidence and guidelines.<br>• Cost-effectiveness	QUAL= 5<br>100%
Roberts et al., 2019/ UK	Semi-structured interview	Cancer nurse specialists (CNS) caring for breast, prostate, or colorectal cancer survivors	Current physical activity promotion practices<br><br>Role in promoting physical activity.	Physical activity promotion	n=19	CNSs stated that discussing and supporting PA with cancer survivors was an accepted and key part of their role.<br><br>CNSs felt they are in a good position to	Discussing physical activity with cancer survivors was a key part of their role.	1. Lack of accurate knowledge of the physical activity guidelines.<br><br>2. Time constraints<br><br>3. Lack of resources.	Cancer nurses play an important role in physical activity promotion in cancer care.	3 themes with 6 subthemes: provision of dietary and physical activity advice, barriers and facilitators.<br><br>• Relationships<br>• Strength of evidence<br>• Lack of knowledge	QUAL= 5<br>100%

			Challenges in physical activity promotion.			inform cancer survivors about PA, signpost and refer to further PA support and continue to promote and support PA throughout treatment and follow-up.<br><br>CNSs perceived that other HPs may be less likely to promote PA.				• Safety concerns<br>• Lack of referrals<br>• Time constraints/resources	
Waterland et al., 2020/ Australia	Semi-structured interviews	GPs	Views on diet and exercise recommendations.<br><br>Experiences of providing dietary and exercise advice.<br><br>Experiences of referring cancer survivors for dietary and exercise services.	Dietary and exercise advice	n=23	Most participants acknowledged importance of exercise and nutrition recommendations for cancer survivors.<br><br>Discussing exercise and nutrition was rarely the reason for a cancer survivor's GP visit so difficult to weave in. GPs perceived the provision of nutrition and exercise advice was part of their role.	1. Time constraints in general practice setting.<br><br>2. Lack of knowledge or experience.<br><br>3. Lack of access to cancer survivors during treatment.<br><br>4. Lack of communication between the tertiary and primary care settings.<br><br>5. Lack of GP-specific resources and programs	GPs value their involvement in supporting healthy behaviours of cancer survivors.<br><br>Future research should tailor exercise and nutrition resources for use within the primary care setting, enhance communication pathways between tertiary and primary care settings and improving access to dietary and exercise professionals.		5 themes with 5 subthemes: benefits of diet and exercise, provision of dietary/physical activity advice, patient preferences, barriers and facilitators.<br><br>• Lack of GP-specific resources<br>• Lack of knowledge<br>• Time constraints<br>• Lack of communication<br>• Referrals	QUAL= 5 100%
**Quantitative studies**											
Alderman et al., 2020/ Australia	Cross-sectional study	GPs	Knowledge, attitudes, perceived behaviours regarding	Physical activity promotion	n=111	GPs agreed PA can improve QOL (92.8%), reduce negative cancer side	GPs are aware that physical activity is safe for people living with cancer and	1.Few GPs had received training regarding physical activity, so they	GPs reported positive attitudes and perceptions towards	3 themes: provision of physical activity advice, barriers, and facilitators.	QUAL= 4 80%

			physical activity.<br><br>GP recommendation and referral rates of physical activity.			effects (89.2%) and saw PA as important and beneficial (91%) to a cancer survivor's journey.<br><br>64% of GPs thought cancer survivors would follow PA advice they provided and 27% of participants reported that cancer survivors asked them about PA.<br><br>Most GPs thought providing PA recommendations was easy (67%), were confident in providing PA recommendations (77.5%). 53.2% believed it was their role to discuss PA cancer survivors.	that believe they play a role in encouraging physical activity.<br><br>GPs prefer exercise physiologists and physiotherapists to deliver physical activity guidance and services.	had to complete additional training.<br><br>2.Not all GPs were aware of the COSA guidelines and had limited access to resources.<br><br>3.GPs had limited awareness regarding physical activity services for people living with cancer.	promoting physical activity for people living with cancer.<br><br>GPs are willing to promote and refer cancer survivors for physical activity.		

Chan et al., 2018/ Australia	Cross-sectional survey	Cancer nurses caring for haematological cancer survivors.	**Perception of responsibility** (5-point Likert; 1=total disagree, 5=total agree) **Confidence in delivering care** (0=cannot do, 10=highly certain can do) **Frequency of care provision (**5-point Likert; 1=never, 5= all the time)<br><br>Barriers for quality survivorship care provision to cancer survivors and for caregivers and/or family members.	Survivorship advice	n=310	**Responsibility** Participants agreed that discussing exercise and physical activity (M=4.48, SD=0.80); health diet recommendations (M=4.40, SD=0.83); and information on health behaviours (M=4.42, SD=0.84), were part of their role. **Confidence** Participants were relatively confident they could discuss exercise and physical activity (M=7.69, SD=2.18); diet (M=7.79, SD=2.09); and information of healthy behaviours (M=7.76, SD=2.13) with cancer survivors. **Frequency** Participants often discussed exercise and physical activity (M=3.31, SD=1.12); diet (M=3.24, SD=1.15); and	Nurses perceived their roles to involve discussing exercise and physical activity, healthy diet and healthy behaviours.	1.Time constraints	There are variations in practice and confidence with respect to implementation of survivorship care practices.	3 themes: provision of physical activity advice, barriers, and facilitators.	QUAL= 4 80%

						healthy behaviour (M=3.05, SD=1.16) recommendations with cancer survivors.					
Haussmann et al., 2018b/ Germany	Cross sectional study	Physicians (GPs, oncologists, surgeons, gynaecologist, urologist, gastroenterologist) & oncology nurses in outpatient and inpatient care.	Demographic information.<br><br>Physical activity promotion behaviour.<br><br>Structural barriers.<br><br>Interest in information resources about physical activity.	Physical activity promotion	n=917	PA was recommended often or routinely in 88.5% of physicians working in outpatient care, 78.1% of physicians working in inpatient care, and 73.1% of oncology nurses.<br><br>Only three HPs indicated that they advised against doing PA.	Health professionals are likely to recommend physical activity to cancer survivors.	1.Time constraints<br><br>2.Physicians in inpatient care who were unable to consult an expert contact person were less likely to promote physical activity to cancer survivors.<br><br>3.Structural barriers (i.e., lack of therapeutic programs; information for cancer survivors and medical staff; specific guidelines; reimbursement for PA counselling, expert contact person).	Most health professionals promoted physical activity to cancer survivors.<br><br>Oncology nurses faced more barriers in promoting physical activity than physicians.<br><br>Most participants, including physicians in outpatient care highlighted the need for information resources.	3 themes: provision of physical activity advice, barriers, and facilitators.	QUAL= 4<br>80%

									Most health professionals frequently promoted physical activity, but structural barriers still exist and differ between professional groups.		
Keogh et al., 2017/ Australia & New Zealand	Cross-sectional survey	Oncology nurses	Physical activity promotion practices.<br><br>Physical activity beliefs.	Physical activity promotion	n=119	Most oncology nurses considered themselves the primary professional group for promoting PA to cancer survivors (n=52/97; 54%) as opposed to a physiotherapist, oncologist, exercise physiologist, other, or "I don't know".<br><br>76% of oncology nurses believed the level of evidence supporting the benefits of PA promotion to cancer survivors was strong.<br><br>Most oncology nurses believed that PA had many benefits for cancer	Oncology nurses perceived their role to involve providing physical activity advice to cancer survivors.	1. Lack of time<br><br>2. Lack of adequate support structures<br><br>3. Lack of expertise and knowledge, and risk to cancer survivors.<br><br>4. Nurses have more frequent opportunities to interact with cancer survivors than oncologists or exercise professionals.	Despite numerous barriers, oncology nurses prefer to promote physical activity across multiple treatment stages because they believe physical activity is beneficial for cancer survivors.<br><br>Hospitals need to provide better support to oncology nurses in promoting PA to cancer survivors.<br><br>There also needs to be better referral pathways to exercise physiologists and physiotherapist s.	3 themes: provision of physical activity advice, barriers, and facilitators.	QUAL= 4<br>80%

						survivors. Nurses agreed or strongly agreed that PA could improve cancer survivors' QOL (n = 107, 90%), mental health (n = 106, 89%), activities of daily living (n = 106, 89%), and reduce risk of developing other chronic diseases (n = 101, 85%).					
Kiss et al., 2020/ Australia	Cross-sectional survey	Dietitian, exercise physiologist, nurses, nutrition/allied health assistant, medical team, physiotherapist, other allied health professionals.	Awareness and understanding of malnutrition and sarcopenia<br><br>Perception of responsibility<br><br>Confidence in malnutrition identification and appropriate management<br><br>Barriers and enablers to identification and appropriate management.	Dietary advice	n=111	84% of participants agreed or strongly agreed it was part of their role to recognise if a cancer survivor was malnourished or sarcopenic and initiate appropriate management.<br><br>Most cancer survivors believed the responsibility for identifying cancer survivors with malnutrition or sarcopenia lay with all health professionals (60%), followed by the medical team (40%) and dietitians	Most health professionals perceived the identification of malnutrition and sarcopenia to be a part of their role.	1. Access to resources<br><br>2. Lack of confidence<br><br>3.Time constraints<br><br>4. Protocols available to support practice and training.	Most health professionals had a good understanding of malnutrition and sarcopenia, as well as their significance in cancer care.<br><br>There were many barriers to delivering optimal nutrition care.	3 themes: provision of diet advice, barriers, and facilitators.	QUAL= 4<br>80%

						(37%).<br><br>90% of participants reported discussing nutrition with cancer survivors.					
Ligibel et al., 2019/ USA	Cross-sectional study	Oncologists, physicians, nurses, physician assistants	Oncology provider practice patterns.<br><br>Perceptions of obesity and weight management during and after active cancer treatment.	Weight management, physical activity, and diet	n=971	89% of participants agreed or strongly agreed that addressing a cancer survivor's weight if overweight or obese should be a standard part of cancer treatment.<br><br>79% believed that the treating physician is responsible for recommending weight loss, increased physical activity, and improved nutrition.<br><br>84% believed that interventions to implement changes in weight, diet, or activity should be conducted by other clinical staff with relevant expertise.	Most health professionals reported advising cancer survivors to maintain a healthy weight/lose weight if overweight, increase physical activity; and eat a healthy, balanced diet during active treatment.	1.Lack of awareness of lifestyle guidelines for cancer survivors.<br><br>2.Lack of training on obesity-related topics.<br><br>3.Time constraint during clinic visits.<br><br>3.Lack of referral programs or specialists.	Most health professionals believed that there was strong evidence linking obesity to cancer outcomes.<br><br>Referrals to providers and programs/services occurred less frequently.<br><br>Further research is needed to support education and training of health professionals to facilitate referrals and overcome barriers.	2 themes: provision of diet and physical activity advice, barriers.	QUAL= 4<br>80%
Puhringer et al.,	Cross-sectional	RNs	Nurses' healthy eating	Nutrition promotion	n=123	Most cancer nurses	Most (35.9%) cancer nurses	1.Time constraints	Most RNs promoted	2 themes:	QUAL= 4

2015/ Australia & New Zealand			promotion habits and beliefs on health eating.			agreed or strongly agreed that healthy eating improved QOL (85.4%), weight management (82.9%), mental health (80.5%), activities of daily living (79.7%) and reduces the risk of other chronic diseases (79.7%) for cancer survivors.<br><br>52.8% promoted healthy eating to cancer survivors during all cancer stages (pre-, during, and post-treatment	considered the dietitian/nutritionist, the primary person responsible for providing healthy eating advice to cancer survivors.<br><br>However, some (32.5%) nurses considered themselves the main person responsible for addressing cancer survivors' nutrition concerns.	2. Lack of adequate support structures.<br><br>3.Lack of nutrition expertise.	healthy eating prior, during and post treatment, as they felt healthy eating has many benefits for cancer survivors.<br><br>Despite favourable attitudes towards promoting healthy eating to cancer survivors across multiple treatment stages, several barriers to healthy eating promotion were reported.	provision of diet advice, barriers.	80%
Spellmann et al., 2014/ Australia	Cross-sectional study	Radiation oncologists, urologists, nurses, medical oncologists caring for prostate cancer survivors	Clinicians' knowledge, attitudes, and practices towards promoting physical activity to prostate cancer survivors.	Physical activity promotion	n=31	54.9% of participants felt that discussing physical activity was part of their role.<br><br>**Knowledge:** Most clinicians (93.6%) strongly agreed or agreed that regular physical activity can improve the	Many medical and nursing health professionals reported that advising cancer survivors on physical activity was not part of their role.	1. Lack of confidence<br><br>2. Lack of resources<br><br>3. Lack of formal training.<br><br>4. Lack of referrals to an exercise professional due to health professionals being unaware of	Despite having a good understanding on the importance of physical activity, few health professionals provided physical activity advice (e.g., was always verbal).<br><br>No health professionals	2 themes: provision of physical activity advice, barriers.	QUAL= 4<br>80%

						quality of life of cancer survivors.<br>50% strongly agreed or agreed that regular physical activity was associated with reduced side effects of treatment.<br>**Confidence:** 83.9% agreed and strongly agreed they were confident to provide physical activity advice.<br>**Practices (advice regarding physical activity):** Only 3.2% of participants always gave this advice; 36% often gave advice, 45.2% sometimes gave advice and 16% rarely gave advice		exercise professionals.	reported that they referred cancer survivors to an exercise professional.<br><br>Physical activity advice may not be provided frequently. Further research should address this issue.		
Wallace et al., 2015/ Australia	Cross-sectional study	Oncology nurses caring for cancer survivors with haematological cancer	Perspectives, confidence levels, and delivery of survivorship practices<br><br>Barriers influencing survivorship practices	Survivorship advice	n=119	Most oncology nurses agreed discussing exercise and physical activity (85%), healthy diet recommendations (82%), and health behaviours (80%) was part of the nursing role.<br><br>Most nurses believed that discussing exercise, physical activity and healthy diet was a nursing role.		1. Lack of time<br><br>2. Limited educational resources.<br><br>3. Lack of dedicated end-of-treatment consultation and insufficient skills/knowledge.	Cancer centres should implement an appropriate model of survivorship care and provide improved training and educational resources for nurses to	2 themes: provision of diet and physical activity advice, barriers.	QUAL=<br>4<br>80%

									enable them to deliver quality survivorship care and meet the needs of haematological cancer survivors.		
Williams et al., 2013/ UK	Cross sectional study	Cancer survivors & individuals in a cancer survivor’s social network.	Attitudes towards advice on diet and physical activity.	Health behaviour advice (physical activity, healthy eating, weight loss)	n=222	Most cancer survivors (87–93%) agreed that advice on diet, activity and weight would be ‘beneficial’, ‘helpful’ and ‘encouraging’,<br><br>Most cancer survivors (84–87%) thought it was ‘the doctor’s duty’ to provide advice on physical activity, diet, and weight loss.	Most cancer survivors found lifestyle advice helpful and believed that doctors had a duty to provide it.	1. Cancer survivors believe health behaviour advice to be beneficial so are more likely to adopt these behaviours.	Not only do most cancer survivors welcome advice on diet, activity and weight, but their family and friends are also likely to be supportive.		QUAL=4<br>80%
**Mixed methods studies**											
Anderson et al., 2013/ UK	Mixed methods (questionnaire & telephone interviews	Doctors and nurses working in colorectal cancer (consultant surgeons & oncologists, surgical registrars, nursing staff)	Familiarity with providing lifestyle advice. Body weight-recording, recognition. Access to support services. Perceived importance of lifestyle changes.	Weight management	n=323	53% of participants were familiar with lifestyle advice guidance for cancer survivors.<br><br>89% indicated weight management was important for improving cancer survivor health.	Discussing weight management was perceived to be the role of the GP, multidisciplinary team, clinical nurse specialist, consultant, and physiotherapist.	1.There is limited evidence on the impact of weight management.<br><br>2. Doctors did not want to affect the patient-doctor/nurse relationship by raising difficult topics (i.e., weight management).<br><br>3. Time constraints	Lifestyle changes in obese colorectal cancer survivors are perceived to be beneficial.<br><br>Written advice tailored to colorectal cancer survivors may encourage them to follow healthier lifestyles.	3 main themes with 3 subthemes: benefits of diet and exercise, HP’s concerns, and barriers.<br><br>• Relationships<br>• Time constraints<br>• Lack of training	MM=4<br>80%

						26% said they were 'always aware' and 36% said they were 'often aware' of BMI.<br><br>47% had referred some overweight cancer survivors to other service providers for weight management during the previous month.<br><br>Qualitative data suggested body weight was not routinely measured at follow-up clinics and that clinicians' concerns about body weight were principally related to identifying weight loss as an indicator of recurrence.		4. Lack of training on weight management.	Health professionals consider weight management important in colorectal cancer survivors.<br><br>However, current perceptions, knowledge and skills suggest further training is required.		
Kassianos et al., 2017/<br>UK	Mixed methods (cross sectional survey and open-ended questions)	GPs caring for prostate cancer survivors	**Primary outcomes:**<br><br>Dietary behaviour change (general)<br>Dietary behaviour change (specific)	Promote dietary changes	n=95	GPs felt that they were the medical speciality mainly responsible for providing dietary-related information<br><br>GPs sceptical on the benefits	GPs are considered to have a role in providing dietary-related information to cancer survivors.	1. Lack of knowledge about diet in relation to cancer.<br><br>2. Lack of confidence.<br><br>3. Lack of information/resources is needed regarding cancer	GPs are interested in promoting dietary changes but do not have the relevant knowledge and confidence.<br><br>Dietary interventions should consider	3 main themes with 3 subthemes: benefits of diet and exercise, provision of dietary/physical activity advice, and barriers.<br><br>• Evidence<br>• Lack of knowledge & confidence	MM=4<br>80%

			**Secondary outcome:**<br><br>Dietary behaviour change (specific)			of dietary changes post-diagnosis and did not want to provide information post-diagnosis to avoid raising false hopes.<br><br>GPs reported lack of confidence in recommending dietary changes. Visual aids (i.e., leaflets) were recommended to help inform cancer survivors.		survivors' awareness, self-care, and diet information.	cancer survivors' cognitive ability, relationship with their health professionals and wellbeing.	• Lack of information	

List of acronyms: BMI=body mass index, GP= general practitioner, HP= health professional, PA= physical activity, PCP= primary care physician, QOL=quality of life
